# Anticancer properties of ester derivatives of betulin in human metastatic melanoma cells (Me-45)

**DOI:** 10.1186/s12935-016-0369-3

**Published:** 2017-01-03

**Authors:** Małgorzata Drąg-Zalesińska, Marcin Drąg, Marcin Poręba, Sylwia Borska, Julita Kulbacka, Jolanta Saczko

**Affiliations:** 1 Department of Histology and Embryology, Wroclaw Medical University, Chałubińskiego 6a, 50-368 Wroclaw, Poland; 2 Department of Medical Biochemistry, Wroclaw Medical University, Chałubińskiego 10, 50-368 Wroclaw, Poland; 3 Department of Bioorganic Chemistry, Department Faculty of Chemistry, Wroclaw University of Science and Technology, Wyb. Wyspiańskiego 27, 50-368 Wroclaw, Poland

**Keywords:** Human melanoma, Betulin derivatives, Cytotoxicity, Apoptosis

## Abstract

**Background:**

Betulinic acid and betulin are triterpenes that have anticancer properties in various types of cancer. Unfortunately, the bioavailability and the bio-distribution of betulinic acid and its metabolic precursor, betulin are very low because of poor solubility in aqueous buffers.

**Methods:**

In this study, we examined the anticancer properties of the ester derivatives of betulin compared to their precursors in a malignant melanoma cell line. We assessed five amino acid esters of betulin. The compounds contained four basic amino acids—natural lysine (l-Lys-OH) and three of its derivatives (l-Dap-OH, l-Dab-OH, and l-Orn-OH)—and alanine (l-Ala-OH) as a negative control (amino acid without an amine group in the side chain). The derivatives were more soluble than their precursors (betulin and betulinic acid) in water. The betulin esters were tested in the malignant melanoma cell line Me-45. To evaluate the cytotoxicity, MTT test was performed after 24, 48 and 72 h of incubation with the test compounds at a concentration range of 0.75–100 μM. For analysis of the apoptotic activity, TUNEL assay was performed. Additionally, expression of caspase-3 and PARP-1 was investigated immunocytochemically.

**Results:**

The highest biological activity was observed with the lysine ester. The results showed that the highest cytotoxicity and the highest number of positively stained nuclei in metastatic melanoma Me-45 cells were obtained after 72 h of incubation with betulin derivatives containing lysine and ornithine.

**Conclusions:**

The betulin ester derivatives showed enhanced antitumor activity compared to their non-modified precursors. Esters of betulin can be more potent anticancer agents than their precursor as a consequence of the rapid bioavailability and increased concentration in cancer cells.

## Background

Cancers are a group of diseases that involve modified cells with dysfunctional proliferation. Melanoma is the most dangerous type of skin cancer observed in clinical practice, and its incidence has increased over the past 40 years at a faster rate than any other type of cancer. Melanoma has a high mortality rate because of its prominent metastasis and its resistance to chemo- and radiotherapy. Currently, the standard anti-melanoma therapy is based on surgical procedures and chemo- and immunotherapy, but these methods are still ineffective. Many new anticancer drugs have been developed to treat this type of cancer. One class of drugs is the triterpenes. These compounds are abundant in natural sources and belong to a group of isoprenoids. They have a wide variety of pharmacological activities [1]. We focused on triterpenes derived from the bark of the birch: betulin and betulinic acid (the oxidation product of betulin) that showed anticancer properties. Previous in vitro and in vivo studies have demonstrated that betulin and betulinic acid were cytotoxic and induced apoptosis in various tumor cells [2–4]. Another report showed that betulinic acid was an extremely selective compound, which was effective in human melanoma cells in vitro and in a mouse model [5]. Moreover, these substances can induce apoptosis in different type of cancer cells by activation of the intrinsic apoptotic pathway [6, 7]. Unfortunately, both betulin and its oxidation product have very poor solubility in aqueous media, and consequently, their bio-distribution and bioavailability are limited in medical applications. However, betulin is a natural compound that is easily isolated from plant material and can be easily converted into more soluble derivatives [8]. Betulin derivatives were previously shown to have increased solubility in water [9]. Our group synthesized five amino acid esters of betulin from the bark of birch (National patent application No PL211589). First, the cytotoxicity of these derivatives was investigated using human epidermoid carcinoma cells (A431) and normal human keratinocytes (HaCaT). The promising anticancer activity and low toxicity towards normal human keratinocytes of these esters suggested that these compounds are promising candidates for further research. Additionally, because no successful treatment for melanoma is currently available, we studied the effects of the new derivatives of betulin on a melanoma cell line.

## Methods

### Cells and culture conditions

A human pigmented malignant melanoma cell line was used (Me-45). This cell line was established in 1997 at the Radiobiology Department of the Center of Oncology in Gliwice, Poland (gift from Prof. M. Latocha) from a lymph node metastasis of skin melanoma from a 35-year-old female patient. The cells were grown in DMEM (Sigma) with 10% fetal bovine serum (Sigma-Aldrich) and antibiotics (antibiotic–antimycotic Stabilized, Sigma). For further studies, the cells were removed by trypsinization (Trypsin–EDTA, Sigma) and washed with PBS. The cells were grown in a humidified atmosphere at 37 °C and 5% CO_2_.

### Betulin and betulin derivatives exposition

**Fig. 1:**
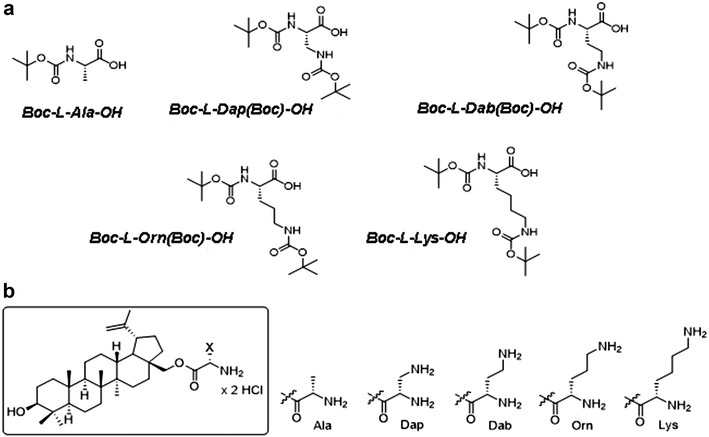
Structures of betulin derivatives: **a** structure of amino acids used for the synthesis of betulin derivatives used for the treatment of melanoma cells (Me-45); **b** the general structure of the 5-element collection containing l-amino acids

In this study, we used betulin and betulin derivatives for metastatic melanoma treatment. The structures of the compounds are shown in Fig. 1. Synthesis of these compounds was described in our previous study by Drag-Zalesińska et al. [10]. The experimental conditions were based on our previous reports. For our studies, we used five selected betulin ester derivatives: four basic amino acids—natural lysine (l-Lys-OH) and three of its derivatives (l-Dap-OH, l-Dab-OH, and l-Orn-OH)—and alanine (l-Ala-OH) as a negative control (amino acid without amine group in side chain) [10], as well as betulin and betulinic acid. The derivatives had increased solubility in water compared to their precursors, which was described in our earlier studies [9]. Betulin-Ala-NH_2_ showed no significant inhibition of cellular proliferation. This result was expected because Ala is hydrophobic and does not have an amino group in the side chain, which influences the biological properties of tested derivatives. Based on our previous studies [10], 6 and 12.5 µM were selected as the concentrations of all compounds for further investigation.

### MTT assay

For determination of cell viability with betulin and its derivatives, MTT assay (Sigma, Germany; In Vitro Toxicology assay) was used. The cells were grown on 96-well microplates and incubated overnight to allow for attachment. The melanoma cells were incubated in all compounds for 24, 48 and 72 h at concentrations of 0.75–100 µM. The MTT cytotoxicity assay is based on the colored reaction of tetrazolium salt and assesses the mitochondrial activity of the cells. Mitochondrial function was expressed as the percentage of the viable treated cells compared to the untreated control cells (without compounds and treated separately with DMSO). All experiments were independently repeated three times.

### Immunocytochemical evaluation of PARP-1 and caspase-3

The apoptotic activity was assessed by immunocytochemical determination of caspase 3 and PARP-1. Immunocytochemistry analysis was performed using the ABC method. Cultures were harvested on 8-well microscopic slides (Thermo Scientific). After 24 h of exposure to betulin and betulin derivatives at concentrations of 6 and 12.5 µM, cells were fixed and dehydrated using 4% paraformaldehyde for 10 min. Next, an ethanol gradient was applied. The samples were then permeabilized and blocked by incubation with 0.1% Triton X-100 (Sigma) in PBS. The proteins were visualized with a polyclonal mouse monoclonal antibody (1:100, anti-CASP-3 and PARP-1; Santa Cruz, USA Biotechnology). For conventional bright-field microscopy (peroxidase-ABC labeling), the samples were incubated with a diaminobenzidine-H_2_O_2_ mixture (DAKO) to visualize the peroxidase label and counterstained with hematoxylin (Alchem, Poland) for 30 s. The samples were analyzed with an upright light microscope (Olympus BX51, Japan). Stained cell numbers were evaluated by counting 100 cells in 3 randomly selected fields. The analysis was carried out by two independent investigators. The results were judged to be positive if staining was observed in more than 5% of the cells. The intensity of immunohistochemical staining was evaluated as follows: (−) negative, (no reaction), (+) weak, (++) moderate, and (+++) strong. All experiments were repeated three times.

### TUNEL assay

DNA fragmentation was evaluated using an ApopTag1 (Qbiogene) kit. In this method, the enzyme terminal deoxynucleotidyl transferase labels the 3-OH ends of DNA generated during apoptosis with biotinylated nucleotides. Immunoperoxidase staining was used to detect these fragments. The apoptosis detection kit enables distinction of apoptosis from necrosis by specifically detecting the DNA cleavage and chromatin condensation associated with apoptosis. Human melanoma cells were cultured on 8-well slides (20,000 cells per slide, Roth Germany) overnight for attachment. Then, the cells were treated with the investigated compounds at concentrations of 6 and 12.5 µM for 24 h. After treatment, the cells were fixed with 4% formalin in PBS for 10 min and permeabilized with 0.2% Triton X-100 in PBS for 5 min at room temperature. TUNEL assay was carried out according the manufacturer’s (Millipore) instructions. Nuclei were stained with hematoxylin. Then samples were mounted by DPX (Thermo Fisher Scientific, Germany) on glass slides. Cells with stained nuclei were investigated by counting 100 cells in 3 randomly selected fields. The analyses were performed by two independent investigators. Samples were evaluated with a BX51 upright light microscope (Olympus, Japan).

### Statistical analysis

The significance of differences between the mean values of group of cells exposed to betulin, its derivatives and not treated control cells was assessed with test-T, which returns the probability associated with Student’s *t* test, which was carried out for each experiment individually and n ≥ 3. P ≤ 0.05 (*) was regarded as statistically significant.

## Results and discussion

**Fig. 2:**
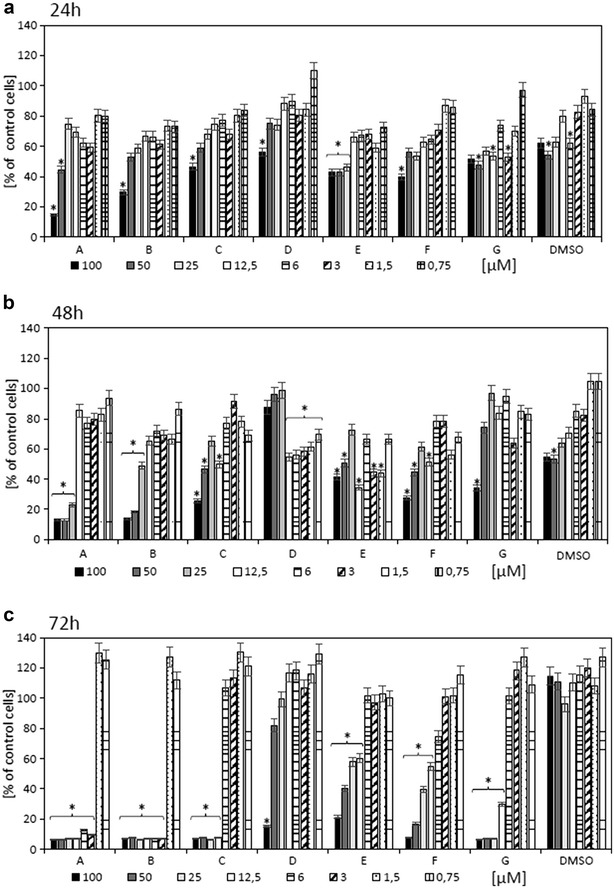
The cytotoxicity studies evaluated Me45 cells treated with betulin and its new derivatives after **a** 24 h; **b** 48 h and **c** 72 h where: *A*—Betulin‐*l* *‐Lys*‐NH_2_; *B—*Betulin‐*l* *‐Orn*‐NH_2_; *C*—Betulin‐*l* *‐Dab*‐NH_2_; *D—*Betulin‐*l* *‐Dap*‐NH_2_; *E—*Betulin‐*l* *‐Ala*‐NH_2_; *F—*betulin; *G—*betulinic acid. P ≤ 0.05 (*)

The resistance of many malignant cells to tumor treatment is a major problem in cancer therapy. Novel strategies are required to improve patient outcomes. Currently applied anticancer therapies are developed for inducing apoptosis or repair defects in the apoptotic pathway in cancer cells. Moreover, many cancer cells are resistant to apoptosis due to mutations in genes responsible for apoptotic death pathways [11]. Numerous anticancer strategies are based on inhibition of cell proliferation and promotion of cell death [12]. Natural compounds derived from plants are frequently used as potential anticancer substances [13, 14]; these compounds are easily available because of their common occurrence in nature. Our results obtained from proliferation assay indicated that the highest cytotoxicity in metastatic Me-45 melanoma cells was obtained after 72 h incubation with new betulin derivatives containing lysine and ornithine (IC_50_ = 2.456 μM; IC_50_ = 2.465 μM, respectively). These results were also observed for the Betulin-l-Dab-NH_2_ compound (IC_50_ = 9.253 μM) compared to the precursors betulin and betulinic acid (Fig. 2; Table 1). The available data indicate that triterpenes are a promising group of anticancer and chemopreventive agents [7, 11]. The most popular compounds from this group are betulin and betulinic acid. Numerous studies have shown that betulin and betulinic acid exhibit biological and pharmacological properties, including anticancer and chemopreventive activities [1, 8]. Additionally, they do not affect normal cells [8, 15, 16]. Unfortunately their biological properties are limited because of the poor water solubility [2]. Betulin from birch bark was modified by our research group with selected natural amino acids (national patent application, No PL211589), which had increased water solubility compared to that of betulin and betulinic acid. Our previous data confirmed that the new compounds have cytotoxic properties and proapoptotic activity in cancer cells [9].

**Table 1:** The IC50 index and water solubility of betulin derivatives evaluated for human Me45 cells

Compound	IC_50_ for Me45 cells [µM]			Water solubility
	24 h	48 h	72 h	
Betulin‐*l*‐*Lys*‐NH2	55.339	20.275	2.456	Very good
Betulin‐*l*‐*Orn*‐NH2	70.658	27.155	2.465	Very good
Betulin‐*l* *‐Dab*‐NH2	66.453	51.405	9.253	Very good
Betulin‐*l*‐*Dap*‐NH2	107.740	61.524	75.489	Very good
Betulin‐*l* *‐Ala*‐NH2	86.573	62.089	10.125	Very good
Betulin	24.208	42.822	30.456	Weak
Betulinic acid	22.772	22.794	15.325	Middle

**Fig. 3:**
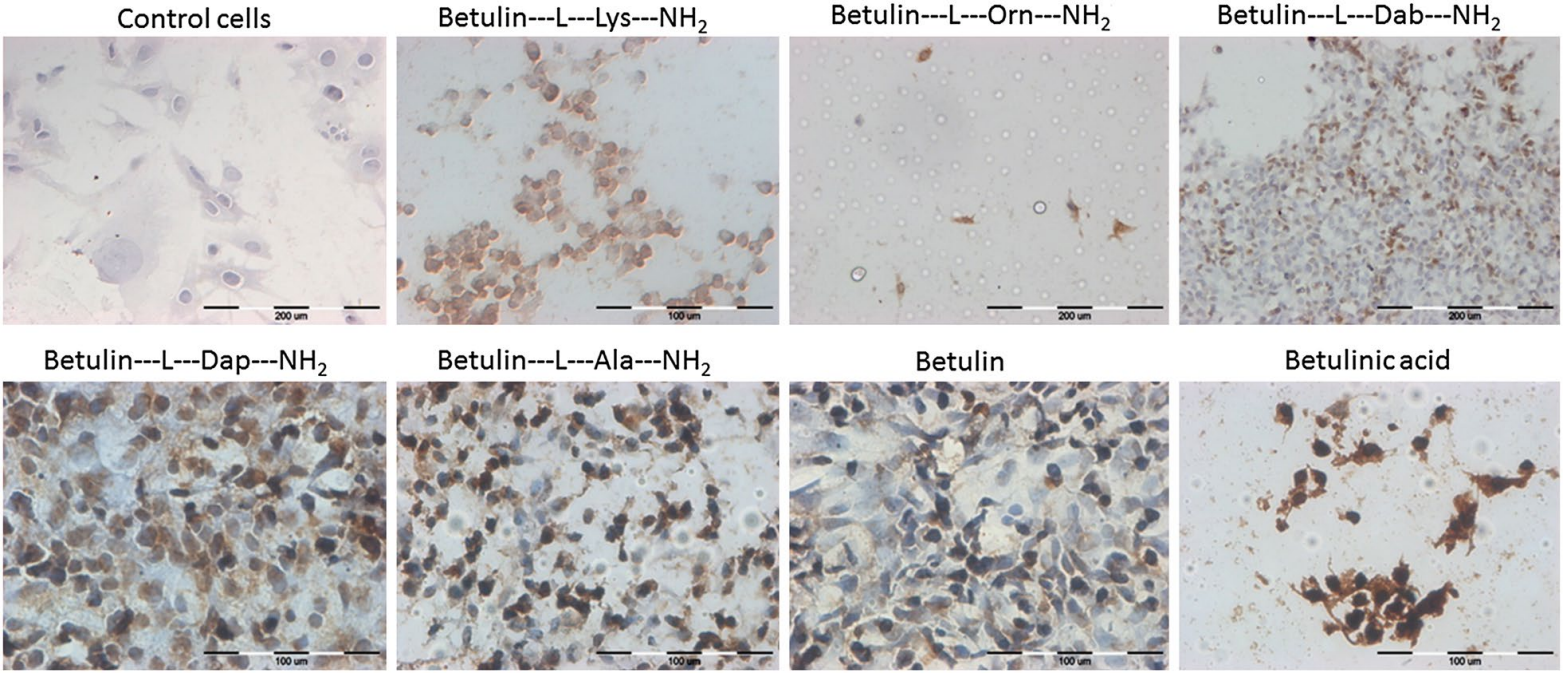
Apoptotic nuclei evaluation in Me45 cells after treatment with betulin and its new derivatives at a concentration of 6 µM (TUNEL assay) 72 h after treatment

In the current study, immunocytochemical staining of PARP-1 and caspase-3 in melanoma cells treated with betulin compounds was performed. PARP-1 is cleaved by caspase-3 through apoptosis, which leads to its inactivation [8, 9]. The results from the immunocytochemical analyses of apoptotic proteins are presented in Table 2. Overexpression of PARP-1 was observed in melanoma cells after exposure to every compound: betulin and its derivatives achieved approximately 100%. Low expression of caspase-3 (<5% for both concentrations) was observed for modified and non-modified substances prepared from birch bark (Table 2). Additionally, TUNEL assay was used to assess the number of apoptotic nuclei after treatment. The highest number of positively stained nuclei was observed in Me-45 cells after incubation with betulin derivatives containing ornithine, lysine, and alanine and betulinic acid (Fig. 3). After incubation with betulin, the lowest number of apoptotic nuclei were noted (Table 3). In other studies, we examined the cytotoxicity and ability of these derivatives to induce apoptosis in skin cancer cells (A431) and normal keratinocytes (HaCaT) [10]. Our investigation suggested that selected derivatives of betulin can be also useful in the treatment of melanoma cells. The highest cytotoxicity was noted in Me-45 cells after incubation with new betulin derivatives containing lysine and ornithine. Similar results were also observed with the Betulin-l-Dab-NH_2_x2HCl compound compared to its precursor betulin. The highest number of positively stained nuclei was obtained in Me-45 cells after incubation with betulin derivatives containing ornithine, lysine and alanine as well as betulinic acid. After incubation with betulin, the lowest number of apoptotic nuclei were noted. In previous investigations, novel lysine esters of betulin were shown to promote apoptosis in tumor-sensitive and resistant pancreatic and stomach cell lines more effectively than betulin and betulinic acid [2, 9]. Orchel et al. examined the effects of betulin and its acetylenic derivative 28-*O*-propynoylbetulin on proliferation and apoptosis in human melanoma cells (G-361). They modified betulin through insertion of a propynol motif into the betulin structure at the C-28 position. The authors compared the cytotoxic and proapoptotic properties of betulin and its derivatives. The investigations demonstrated similar cytotoxicity, inhibition of the cell cycle and apoptosis activation in melanoma cells [7]. Additionally, Boryczka et al. showed that modified betulin at the C-28 position had stronger cytotoxic properties against human and murine leukemia cells than its precursor, an acetylenic derivative and cisplatin. However, 28-*O*-propynoylbetulin had less apoptotic and cytotoxic activity in breast and colorectal cancer cells [17]. Furthermore, Hata et al. [18] noted that the lupine triterpenes showed significantly increased cytotoxicity than betulin in melanoma, leukemia and neuroblastoma cells.

**Table 2:** Semi-quantitative evaluation of immunocytochemical reaction with anti-PARP-1 and anti-caspase 3 antibodies in Me-45 cells after exposition to betulin derivatives, after 24 h

Compound	Me-45			
	PARP-1 12.5 µM	PARP-1 6 µM	Casp 3 12.5 µM	Casp 3 6 µM
Control cells	5%, +/++	–	<5%,∓	–
Betulin‐*l* *‐Lys*‐NH_2_	95%, ++	50%, +/++	0%	0%
Betulin‐*l* *‐Orn*‐NH_2_	100%, ++	98%, +/++	<5%, ∓	<5%, ∓
Betulin‐*l* *‐Dab*‐NH_2_	100%, +++	100%, +++	0%	0%
Betulin‐*l* *‐Dap*‐NH_2_	100%, +++	98%, +/++	<5%, ∓	<5%,∓
Betulin‐*l* *‐Ala*‐NH_2_	100%, ++/+++	90%, ++	<5%, ∓	0%
Betulin	100%, ++/+++	95%, ++/+++	10%,+	<5%,∓
Betulinic acid	100%, ++/+++	100%, ++/+++	5-10%, +/++	<5%, ∓

**Table 3:** Apoptotic nuclei evaluation by TUNEL assay in Me-45 cells after exposition to betulin derivatives in concentration 6 µM in 72 h after treatment

Compound	% of cells with stained nuclei
control cells	0
Betulin‐*l* *‐Lys*‐NH_2_	86
Betulin‐*l* *‐Orn*‐NH_2_	100 (cells damage, reduced number of cells)
Betulin‐*l* *‐Dab*‐NH_2_	34
Betulin‐*l* *‐Dap*‐NH_2_	48
Betulin‐*l* *‐Ala*‐NH_2_	78
Betulin	27
Betulinic acid	95

In our study, treatment of Me-45 melanoma cells with the earlier described derivatives resulted in significant increases in apoptotic nuclei and strong cytotoxicity in these cells compared to cells incubated with betulin and betulinic acid. The immunocytochemical staining showed similar amounts of caspase 3 and PARP-1 after incubation with betulin and its selected derivatives. It is worth noting that we used esters of betulin with different numbers of free amino groups (one or two), which influence their solubility in water. Additionally, the biological properties and activity of these esters of betulin were positively associated with the length of the side chain of the l-amino acid.

In various studies, the cytotoxic and proapoptotic properties of betulin and betulinic acid were demonstrated in different melanoma cells. Moreover, the mechanism of this apoptotic activity was described. Betulin and betulinic acid increased the apoptosis of melanoma cells via the interaction with the mitochondrial membrane and the release of cytochrome c or apoptosis inducing factor (AIF). Importantly, betulinic acid induces apoptosis in cancer cells independent of their p53 status [16, 19–24].

Our results demonstrated that modification of betulin through the addition of selected amino acids can result in stronger cytotoxic and proapototic effects compared to betulin. As shown in our studies, esters of betulin can be more potent anticancer agents than their precursor as a consequence of more rapid availability and increased concentration in cancer cells. The cytotoxic and proapoptotic mechanism of these compounds may be similar to betulin and betulinic acid, but this hypothesis must be further investigated. In a future study, we will examine the mechanism of apoptosis after betulin ester treatment by evaluating the major proteins involved in this type of death. Elucidation of the apoptotic pathway induced after incubation with the analyzed compounds can help in the development of new anticancer strategies.

## Conclusions

We concluded that the betulin ester derivatives efficiently enhanced antitumor activity compared to their non-modified precursors in metastatic melanoma cells. The results indicated that natural compounds derived from betulin can be more potent anticancer agents than its precursor as a consequence of rapid bioavailability and better concentration in skin cancer cells.

## Abbreviations

AIF: apoptosis inducting factor
PARP-1: poly [ADP-ribose] polymerase 1
Me-45: human metastatic melanoma
A431: human epidermoid carcinoma cells
HaCaT: human keratinoctytes
PBS: phosphate-buffered saline
DPX: distrene-80, plasticizer, xylene traditional mounting medium
